# A Novel Self-Assessment Method for Training Access Cavity on 3D Printed Endodontic Models

**DOI:** 10.3390/dj11060152

**Published:** 2023-06-13

**Authors:** Matteo Meglioli, Giovanni Mergoni, Francesco Artioli, Benedetta Ghezzi, Maddalena Manfredi, Guido Maria Macaluso, Simone Lumetti

**Affiliations:** 1 Center of Dental Medicine, Department of Medicine and Surgery, University of Parma, Via Gramsci 14, 43126 Parma, Italy; 2 IMEM-CNR, Parco Area delle Scienze 37/A, 43124 Parma, Italy

**Keywords:** endodontics, access cavity, dental education, 3d printing, intraoral scanner

## Abstract

Background: New technologies can facilitate the transition from pre-clinical to clinical settings. We investigate students’ satisfaction with a novel learning method adopted in access cavity exercises. Methods: Students performed their access cavity on inexpensive, in-house 3D printed teeth. Their performances were evaluated by scanning the prepared teeth with an intraoral scanner and visualized using a mesh processing software. Then, the same software was used to align the tooth prepared by the student and the teacher’s one for self-assessment purposes. Students were asked to answer a questionnaire about their experiences with this new learning method. Results: From the teacher’s perspective, this novel learning approach was easy, straightforward and affordable. Overall, student feedback was positive: 73% found that access cavity assessment by scanning was more useful compared to a visual inspection under magnification and 57% reported that they had a better understanding of errors and mishaps. On the other hand, students pointed out that the material used to print teeth was too soft. Conclusion: The use of in-house 3D printed teeth in pre-clinical training is a simple way to overcome some of the drawbacks associated with extracted teeth, such as limited availability, variability, cross-infection control, and ethical constraints. The use of intraoral scanners and mesh processing software could improve student self-assessment.

## 1. Introduction

The primary goal of root canal treatment is to heal or prevent apical periodontitis [1], a tooth disorder with a high frequency among adults [2]. Since the endodontic specialty is still not established in Italy, root canal treatments are frequently performed by general dentists, who, in many cases, do not reach optimal standards [3,4]. The average quality of endodontic treatments performed by general dentists was also found to be suboptimal in other countries [5,6,7]. A possible cause could be inadequate training during the undergraduate curriculum [8].

Pre-clinical training is a fundamental learning step for dental students to become familiar with the techniques and procedures that they will then apply in a clinical setting [9]. In consideration of the importance of pre-clinical training in dental education, every improvement is strongly encouraged, and new technologies can serve this purpose [10]. A joint statement from the European Society of Endodontology (ESE) and the Association for Dental Education in Europe (ADEE) recommended the development of intelligent systems to facilitate preclinical skills training and to provide instantaneous feedback on performance [11]. 

Extracted teeth have long been used in pre-clinical training in endodontics because they are cost-free, allow students to practice with a range of anatomical variations and pathologies, and provide valuable haptic feedback, allowing students to develop the tactile sensitivity and manual dexterity necessary for successful endodontic procedures [12,13]. According to recent studies, human extracted teeth are used for endodontic training in 82.1%, 73% and 100% of Italian, English and Spanish dental schools, respectively [14,15,16]. Nevertheless, the use of extracted teeth shows some limitations. For example, with the improvement of oral health in the general population and with the new therapeutic possibilities for teeth that were previously destined for extraction, the availability of extracted teeth suitable for practice has decreased [17]. The use of extracted teeth may carry ethical concerns and require preventive measures to avoid the risk of cross-infections [13,18]. In addition, the lack of uniformity of natural teeth poses difficulties during classroom exercises and when student learning is assessed [19].

For these reasons, increasingly sophisticated artificial root canals have been put on the market, from canals of different shapes and dimensions in clear resin blocks to plastic typodonts [20,21]. In particular, some companies are specialized in the production of 3D printed teeth from X-ray tomography or micro-tomography scans of extracted teeth [19]. 

In various medical fields, students can enhance their skills on accurate 3D printed models that reproduce the haptic feedback of the patient’s tissues [22]. Several authors described temporal bone surgeries [23,24], implant treatment, or maxillary sinus floor augmentation [25] training in realistic in vitro conditions using these 3D-printed models.

In the dental field, these models are mostly used by students for preclinical education in caries excavation, direct capping of the pulp, core build-up, and crown preparation [26].

The cost of these replicas depends on the type of tooth and how accurate the anatomical characteristics are. Given the need to simultaneously and repeatedly train multiple students, a large number of replicas must be utilized, making low cost a crucial characteristic of any effective training model [27]. The gradual reduction of 3D printer costs has made it possible to print teeth in-house [28]. The advantages of using 3D printed teeth are the potentially infinite availability of tooth replicas and the elimination of variability of natural teeth [12]. 

Intraoral scanners (IOSs) are widely used tools that allow dentists to create detailed digital impressions [29]. The functioning of these devices is based on a light source, such as a laser or structured light, which is projected onto the object undergoing scanning. Imaging sensors capture the resulting images, which are subsequently processed by the scanning software. This software analyzes the images and generates point clouds, which are collections of points representing the object’s surface. These point clouds are then transformed into a 3D surface model, also known as a mesh, through a process called triangulation [30]. Besides their valuable use in clinical practice, IOSs have shown several possible applications in dental education. Seet et al. assessed the effectiveness of IOSs in student crown preparation evaluation and concluded that they can overcome limitations in conventional assessment of objective parameters and some subjective parameters [31]. Park et al. reported the benefits of a computer-assisted design/computer-assisted manufacturing (CAD/CAM) learning software which allows students to objectively assess their performance in preclinical prosthodontics [32]. Most recently, Choi et al. used IOS as a feedback tool to assess student access cavity on 3D printed teeth [33].

The purpose of this study was to describe a new learning method adopted in the cavity access exercises at Parma dental school.

## 2. Materials and Methods

The project involved the nineteen fifth-year dental students who attended the Endodontics course at Parma University from September 2022 to January 2023. The undergraduate endodontic program consists of 50 h of theory lessons and 75 h of pre-clinical training. Pre-clinical training consists of applying the notions learned during frontal lessons on endodontic treatment, from pulp cavity opening to canal shaping, cleaning, and obturation on extracted teeth under the direct supervision of the tutors. Contrary to previous years, in the course from 2022–2023, the students also practiced on 4 to 5 in-house 3D printed teeth, as well as on a similar number of natural teeth.

**Figure 1:**
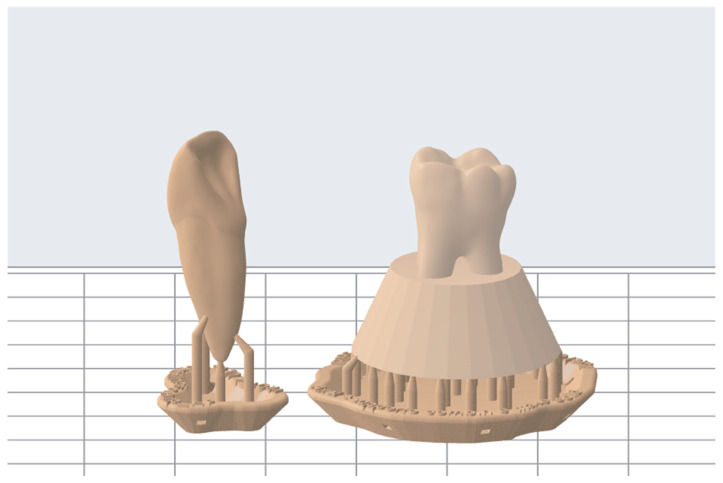
STL files loaded on PreForm software and creation of the 3D printed support.

Replicates of teeth 1.1 and 3.6, reproducing internal and external anatomy, were obtained as follows. STL teeth files were freely available on the web [34]. Using PreForm software (Formlabs, Somerville, MA, USA), STL files were loaded, and an adequate 3D printing support was designed (Figure 1). 

Layer thickness of 100 µm was set. A vat photopolymerization 3D printer (Form 2, Formlabs, Somerville, MA, USA), charged with Model V2 resin (Formlabs, Somerville, MA, USA), was used. This resin, which was fabricated to produce dental master models, was chosen for its mechanical characteristics and accuracy. 

**Figure 2:**
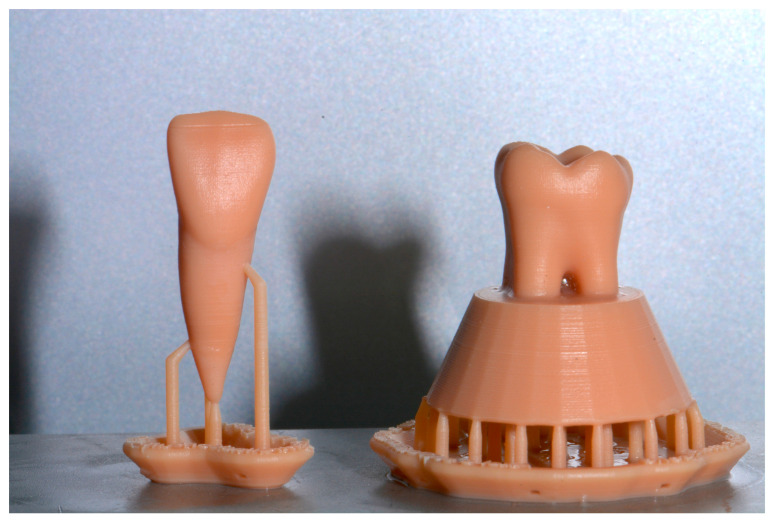
1.1 and 3.6 tooth replicas after post-curing process.

After 3D printing, the post-curing process was performed using Form Wash and Form Cure (Formlabs, Somerville, MA, USA). First, the models were immersed in 97% isopropyl alcohol for 10 min using Form Wash and then polymerized for 80 min at 60 °C using Form Cure. After removing the 3D printing support, the resulting teeth were ready to use (Figure 2).

**Figure 3:**
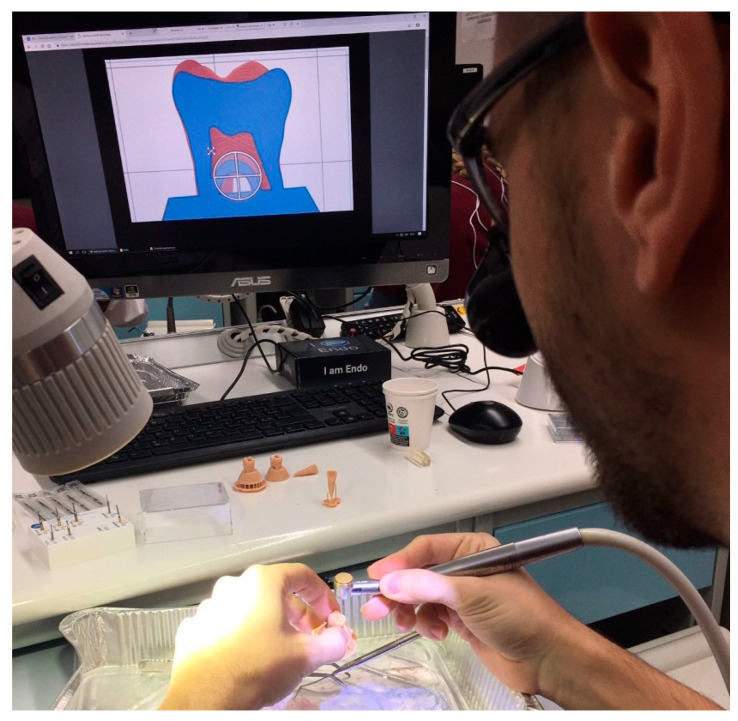
Access cavity exercises on 3D printed teeth during student pre-clinical training.

The students performed access cavities using burs mounted on air-driven and motor-driven handpieces, as usual (Figure 3).

**Figure 4:**
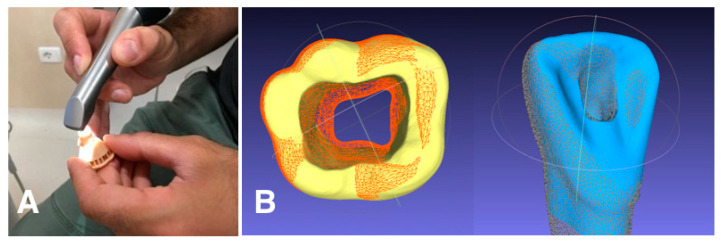
Digital scanning of the prepared tooth (**A**) and visualization via MeshLab software (**B**).

Unlike the training on natural teeth, the students were not under the direct supervision of the tutors during the training on printed teeth. The assessment of the access cavities was carried out as follows. The prepared teeth were scanned with Omnicam (Dentsply Sirona, Charlotte, NC, USA). If any voids or scanning errors were found in the resulting 3D model after each scan, the tooth was rescanned as needed. Scan data were exported from the Omnicam station as STL files and visualized using Meshlab v.2022.02 software (Visual Computing Lab, Pisa, Italy). This open-source software allowed the students to view the prepared tooth by rotating it in space and enlarging the image as desired to better evaluate the size, shape, extent, complete deroofing, convenient form, gouging, and the presence of perforations. Additionally, the software allowed the student to align their model with an ideal access cavity, obtained by scanning a model prepared by the teacher, to make a direct comparison and highlight the differences (Figure 4). Thanks to Meshlab, it was also possible to mathematically evaluate the geometric difference between the scanned teeth, employing a quantitative approach. This was achieved by utilizing a tool known as “Hausdorff distance”. The Hausdorff algorithm is commonly used to assess the dissimilarity or similarity between two sets of points within a metric space [35,36]. By applying the Hausdorff distance, we were able to determine the extent to which the sets of points deviated from each other. Specifically, the Hausdorff distance calculates the maximum distance between any point in one set and its nearest counterpart in the other set, thereby providing a measure of how far apart the sets are. Notably, this distance metric takes into account both directions, considering the longest distance from any point in one set to its closest point in the other set. To quantify the discrepancy between the models created by the students and the ideal model, we calculated the Hausdorff distance values. These values represented the dissimilarity between the two sets of points. Subsequently, we derived the root mean square (RMS) from these Hausdorff distance values. The RMS is a statistical measure that entails calculating the square root of the arithmetic mean of the squares of a given set of values. In simpler terms, it offers a way to determine the average value of a set of numbers, considering both positive and negative values. By employing the RMS, we obtained a comprehensive assessment of the overall disparity between the models. This quantitative analysis provided valuable insights into the extent of variation between the students’ prepared tooth and the teacher’s one.

At the end of the course, and after the approval of the local research ethics board (protocol number 94637/2023), the students’ feedback and satisfaction with the access cavity training were investigated using a voluntary questionnaire. Students were invited to participate through a Google form shared by e-mail. They were informed of the purpose of the survey and that the answers would be treated anonymously, and that the questionnaire would not influence the evaluation of learning. The students were asked about difficulties in obtaining extracted teeth, differences between natural teeth and 3D printed teeth, usefulness of 3D printed teeth in preclinical training, and usefulness of self-assessment using digital scanning and alignment software (See Supplementary Materials). For most of the questions, students had to answer using a 5-point Likert scale (1—strongly disagree, 2—disagree, 3—neutral, 4—agree, 5—strongly agree). In some closed questions, the students could answer “yes/no” or “less/equal/more”. Open-ended questions were asked on the benefits of using natural teeth or artificial teeth and their overall experience with the proposed training program.

## 3. Results

From the teacher’s perspective, this novel learning approach was simple, straightforward and affordable. After preparing the STL files, the printing time was approximately 12 min per tooth. The cost of the resin for each tooth was around 0.54 euros. The time needed for scanning each tooth was approximately 1 min. 

The RMS values obtained by comparing the openings made by the students in the molars with the reference openings had an average of 0.269 ± 0.05 mm (*n* = 44, min = 0.134 mm, max = 0.336 mm). The RMS values obtained by comparing the openings made by the students in the incisors with the reference openings had an average of 0.279 ± 0.07 mm (*n* = 44, min = 0.07 mm, max = 0.396 mm). The RMS values obtained by comparing the openings made by the students in the total of the teeth with the reference openings had an average of 0.274 ± 0.06 mm (*n* = 88, min = 0.07 mm, max = 0.396 mm).

**Figure 5:**
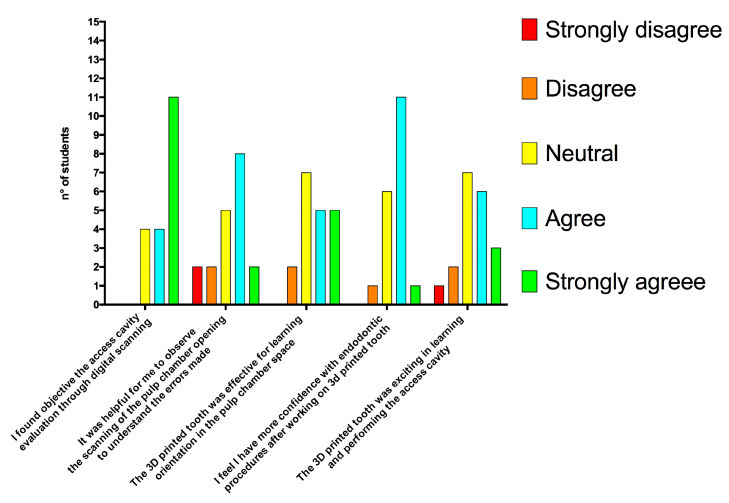
Student feedback after access cavity exercises using in-house 3D printed teeth and digital scanning.

The response rate to the questionnaire was 100% and all the students answered all the questions. Overall, student feedback on the use of 3D in-house printed teeth and intraoral scanning for access cavity training was positive (Figure 5). 

15 (79%) and 14 (74%) agreed with the statements “It was helpful for me to observe the scanning of the pulp chamber opening to understand the errors made” and “I believe that analyzing the pulp chamber opening through digital scanning would be more useful compared to direct inspection”, respectively. Only four students disagreed with the statement “I found objective the access cavity evaluation through digital scanning”. Most of the students (16/19, 84%) found that the 3D printed tooth had a pulp morphology that was easy to understand, and only three students (16%) found it difficult to locate anatomical references for a correct opening in the 3D printed tooth. Most students (11/19, 58%) found 3D printed teeth to be more suitable for the practical exam compared to natural teeth.

**Figure 6:**
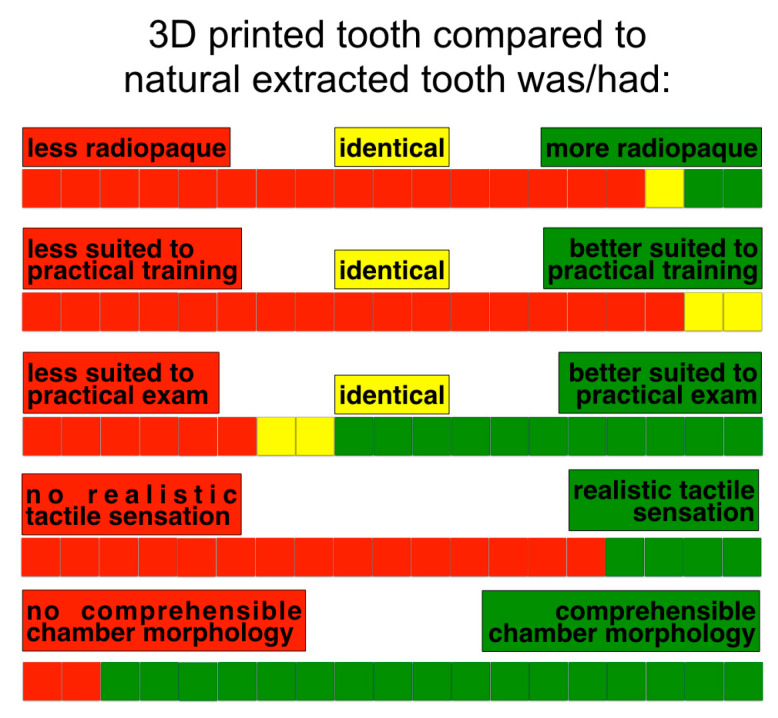
Comparison of the 3D printed teeth and extracted teeth according to the students’ answers.

According to student opinions, the main drawback of printed teeth was the different tactile sensation compared to human dentin (Figure 6). 

All the students found the resin of 3D printed teeth to be softer than the dentin of natural teeth, and 15/19 (79%) stated that the 3D printed teeth did not provide a realistic tactile sensation during preparation. Only seven students (37%) agreed with the statement “The 3D printed tooth is very useful in acquiring fine sensitivity in the use of rotary instruments for the preparation of the pulp chamber access”. A minority of students (6/19, 32%) agreed with the statement “The 3D printed tooth using 3D technology is a valid alternative to the extracted human tooth for training exercises”, and nine students (47%) agreed with the statement “The printed tooth was stimulating in learning and performing the access cavity”. Some students found it difficult to collect extracted teeth (4/19, 21%).

In the free text section of the questionnaire, students reported several advantages of implementing access cavity training with 3D printed teeth. Some students appreciated the higher availability, the absence of cross-infection risk, and the lower risk of fracture of 3D printed teeth compared to natural teeth. The possibility of repeating the exercise on the same model was found useful for perfecting the technique in the early stages of learning, as well as the possibility of using an identical model simultaneously for all trainees. Other students reported that training with a model featuring a standard pulp chamber with no calcification or anatomical variations was advantageous in the initial stages. One student stated that printed teeth allow for a standardized assessment system. Many students suggested, for future courses, increasing the hardness of the resin to offer a better tactile sensation and to prevent the bur from getting stuck. In addition, they demanded other types of 3D printed teeth.

## 4. Discussion

Root canal treatment aims to cure or prevent apical periodontitis [1,37]. To achieve this goal, each stage of the treatment must be carried out properly [38]. The access cavity is one of the most critical technical stages and must be performed with the utmost care and precision. If it is not executed correctly, the subsequent management of the root canal system can be severely compromised [39]. It was reported that the access cavity creation was a stressful procedure for dental students [40] and that it was often associated with iatrogenic procedural errors that could lead to canals being left untreated, scarcely disinfected, and insufficiently shaped and filled [41]. An improper access cavity may lead to the failure of the treatment. For these reasons, dental educators should strive to develop pre-clinical learning methods and new technologies can assist them in this task. 

In this study, we describe a new learning method adopted at the Dental School of Parma for access cavity exercises using in-house 3D printed teeth and an IOS. The use of 3D printed teeth in endodontic education is not a novelty [27,41,42,43]. Indeed, in recent years, different factory-manufactured 3D models have been introduced in the market [19]. Compared to previous artificial teeth, 3D printed ones are more realistic, being capable of faithfully reproducing endodontic anatomy. On the other hand, their high cost puts a limit on their usage in high quantities. The constantly declining costs of in-house 3D printing offers to dental schools the possibility of self-production, in high quantities, of models that are not yet available on the market or are too expensive to purchase [28,34,44,45]. In most cases, the workflow to obtain 3D printed teeth starts with DICOM files of the natural tooth, derived from a CBCT or microCT scan. The DICOM files are then converted into STL files and thoroughly refined, prepared, and smoothed with dedicated software before being printed [46,47]. This type of processing typically requires many hours of work and represents the main limitation of the production process. For practical reasons, in this preliminary project, we decided to use STL files freely available on the internet. Our aim was to verify the feasibility of printing and obtained positive feedback from the students; we planned to produce teeth starting from scans of natural teeth for future Endodontic courses. In addition, in the free text section of the questionnaire, we received a specific request from a student to produce other types of teeth. According to the student’s answers, the 3D printed teeth had a correct pulp morphology and identifiable anatomical references for a conventional access cavity opening, but, similar to what has been reported in other studies [27,48,49,50], the material consistency was not hard enough compared to the dentin of natural teeth. So, our models could not reproduce the tactile perception with rotating instruments. A possible solution was proposed by Robberecht et al., who developed a method for producing ceramic-based models [51,52]. Although the hardness of these artificial root canals, thanks to the use of hydroxyapatite, has been found to be comparable to that of natural teeth, the difficulty and complexity of the production phases prevent their widespread use. The most practical method, which has already partly been pursued by some manufacturers [19], is to develop new resins and advanced post-printing processes capable of obtaining models with mechanical characteristics that overlap with those of natural teeth.

After access cavity training, we used an IOS, available in the clinical area of our dental school, to acquire digital impressions of the prepared teeth. Using MeshLab, a free STL processing software, the scans were viewed by the students with the possibility of enlarging the models and rotating them by 360 degrees. This enables students to observe in detail the tooth preparations. Secondly, the software allowed the superimposition of the student’s model with an ideal cavity access made by the teacher in a previously scanned tooth. In this way, it was possible to instantly highlight the differences, both in excess and in deficiency, in the extension and shape of the cavity. Iatrogenic errors such as incomplete deroofing, gouging or perforations could be easily identified. Using the Hausdorff distance tool included in Meshlab, it was possible to instantaneously and quantitatively quantify the discrepancy between the student and the reference models. We have planned to analyze in the future the accuracy of this value in assessing the quality of access cavity openings. If the RMS value proves to be a valid surrogate for the traditional evaluation by the instructor, it can be used for both self-assessment and formal examination assessment. 

Students’ answers reported high levels of satisfaction with the new self-assessment method. This high approval rating might be due not only to the immediacy of the visual feedback, but also to the generally enthusiastic attitude of dental students towards digital dentistry [53]. Commercial digital evaluation systems, such as PrepCheck^®^ (Dentsply Sirona©, Bensheim, Germany), Dental Teacher™ (KaVo©, Biberach, Germany) and Compare© (Planmeca©, Helsinki, Finland), that report feedback on students’ prosthetic preparations through a comparison with reference preparation scans are already available [54,55,56]. To the best of our knowledge, only one other study used IOS as a learning tool for students in endodontic preclinical training. Choi and colleagues [33] investigated student’s overall experience with a new learning method involving commercial 3D printed teeth, an IOS and a new software, called 3D Dental Aling. Despite some differences between the two protocols (commercial versus in-house 3D printed teeth, Emerald versus Omnicam IOS and 3D Dental Aling versus Meshlab software), both teaching methods received similar positive feedback from students. In our study, we decided to use in-house 3D printed teeth to limit costs and to not have restrictions on the number of teeth available. 3D Dental Align is a custom-developed software based on the 3D Slicer platform. It was designed by the authors to optimize workflows for instructors and students. Aligning the models through Meshlab was more complex, but all the students were able to use it under the instructor’s supervision. We involved Omnicam 2.0 because it was already available in our operative clinical department. A recent study found that Emerald IOS (Planmeca, Helsinki, Finland) was statistically weaker in accuracy and precision than Omnicam [57]. The impact of the IOS reliability on access cavity evaluation should be investigated in future studies.

## 5. Conclusions

The utilization of extracted teeth in dental education comes with inherent limitations, including decreased availability and ethical concerns surrounding their procurement. However, emerging technologies such as 3D printing present an appealing alternative. Several advantages are offered by 3D printed teeth, including enhanced accessibility and the ability to replicate realistic dental structures. By leveraging these technologies, dental educators can overcome the challenges associated with traditional teaching methods.

The results of this study proved that the integration of 3D printing, IOSs and mesh processing software brings further benefits to pre-clinical training and assessment in dental education. This digital approach facilitates the evaluation of student performance in a more efficient and objective manner.

Further research is necessary to fully explore and validate the effectiveness of this innovative learning method when compared to traditional approaches. 

## Data Availability

The data presented in this study are available on request from the corresponding author.

## Supplementary Materials

The following supporting information can be downloaded at: https://www.mdpi.com/article/10.3390/dj11060152/s1, File S1. Questionnaire to assess the new teaching method for access cavity training.
