# Supplementary material for: A Novel Self-Assessment Method for Training Access Cavity on 3D Printed Endodontic Models
Source: Dent J (Basel). 2023 Jun 13;11(6):152. doi: 10.3390/dj11060152 (PMC10297574; doi:10.3390/dj11060152)
Supplement: Supplementary file 1 [file dentistry-11-00152-s001.zip › dentistry-2431765-supplementary.pdf]

ACCESS CAVITY TRAINING – STUDENT QUESTIONNAIRE

Dear student,
we kindly ask you to complete the following questionnaire. Your opinions are valuable for improving our teaching methods. The data will be collected anonymously and will not have any impact on your evaluation. (where 1 to 5 → Please rate this statement on a 5-point scale: 1 - Strongly disagree 2 - Disagree 3 - Neutral 4 - Agree 5 - Strongly agree)

- I had difficulty obtaining the extracted teeth. (1 to 5)

- The 3D printed TOOTH compared to the EXTRACTED TOOTH is:
  - Harder, Same, Softer
  - More radiopaque, Same, Less radiopaque
  - More suitable for practical exercises, Same, Less suitable for practical exercises

- The 3D printed tooth:
  - provides a realistic tactile sensation during preparation / does not provide a realistic tactile sensation during preparation
  - has a comprehensible pulpal morphology / does not have a comprehensible pulpal morphology

- Extracted tooth:
  - is stimulating for learning and performing the access cavity. (1 to 5)
  - is very useful for developing fine sensitivity in the use of rotary instruments for preparing the chamber access. (1 to 5)
  - is effective for learning spatial orientation within the pulp chamber. (1 to 5)

- 3D printed tooth:
  - is stimulating for learning and performing the access cavity. (1 to 5)
  - is very useful for developing fine sensitivity in the use of rotary instruments for preparing the chamber access. (1 to 5)
  - tooth is effective for learning spatial orientation within the pulp chamber. (1 to 5)

- In your opinion, how could 3D printed models be improved for future exercises?

- According to you, what are the advantages of extracted teeth in the context of these exercises?

- According to you, what are the advantages 3D printed teeth in the context of these exercises?

- The 3D printed tooth is a valid alternative to the extracted human tooth for the exercises. (1 to 5)
- I found it difficult to locate the anatomical references for proper access opening in the 3D printed tooth. (1 to 5)
- I feel more confident with endodontic procedures after working on the 3D printed tooth. (1 to 5)
- I find the practical examination on chamber access to be useful and a valuable learning experience. (1 to 5)
- I find the evaluation method using scanning of access cavities in 3D printed teeth to be objective. (1 to 5)
- I found it useful to observe the scanning of the chamber access to understand the errors I made. (1 to 5)
- I find it more useful to analyze the chamber access through 3D scanning or traditional inspection?
